# Endothelial dysfunction in children with type 1 diabetes mellitus

**DOI:** 10.1590/2359-3997000000271

**Published:** 2017-06-14

**Authors:** Antonella Márcia Mercadante de Albuquerque do Nascimento, Inês Jorge Sequeira, Daniel França Vasconcelos, Lenora Gandolfi, iccardo Pratesi, Yanna Karla de Medeiros Nóbrega

**Affiliations:** 1 Universidade de Brasília Campus Universitário Darcy Ribeiro Brasília DF Brasil Programa de Pós-Graduação em Ciências Médicas, Universidade de Brasília (UnB), Campus Universitário Darcy Ribeiro, Brasília, DF, Brasil; 2 Hospital Universitário de Brasília Campus Universitário Darcy Ribeiro Brasília DF Brasil Hospital Universitário de Brasília, Campus Universitário Darcy Ribeiro, Brasília, DF, Brasil; 3 Faculdade de Ciências da Saúde UnB Campus Universitário Darcy Ribeiro Brasília DF Brasil Departamento de Ciências Farmacêuticas, Faculdade de Ciências da Saúde, UnB, Campus Universitário Darcy Ribeiro, Brasília, DF, Brasil; 4 Escola de Ciências e Tecnologia Departamento de Matemática, Matemática e Aplicações Universidade Nova de Lisboa Caparica Lisboa Portugal Escola de Ciências e Tecnologia, Departamento de Matemática, Matemática e Aplicações, Universidade Nova de Lisboa, Quinta da Torre, Caparica, Lisboa, Portugal; 5 Laboratório de Metodologias Aplicadas às Doenças Infecciosas UnB Campus Universitário Darcy Ribeiro Brasília DF Brasil Metodologias Aplicadas, Laboratório de Metodologias Aplicadas às Doenças Infecciosas, UnB, Campus Universitário Darcy Ribeiro, Brasília, DF, Brasil

**Keywords:** Type 1 diabetes mellitus, endothelial dysfunction, atherosclerosis, flow-mediated dilation, carotid intima-media thickness

## Abstract

**Objective:**

The purpose of this study was to verify the presence of endothelial dysfunction and initial structural atherosclerotic changes in children with Type 1 diabetes mellitus (T1DM).

**Subjects and methods:**

The study population comprised 31 diabetic children aged 6 to 12 years, divided into two subgroups according to the duration of the T1DM diagnosis: subgroup 1, with less than 5 years elapsed since diagnosis, and subgroup 2, with more than 5 years elapsed since diagnosis. The control group comprised 58 age-matched healthy children. Ultrasonographic techniques were used to measure the flow-mediated dilatation (FMD) of the brachial artery and the intima-media thickness (IMT) of the carotid arteries.

**Results:**

Children with T1DM with longer disease duration showed significantly decreased mean values of FMD compared with those in the control group. No significant differences between the groups were found in relation to IMT. The FMD percentage presented a moderate negative correlation with glycated hemoglobin (HbA1c) and fasting glucose levels.

**Conclusion:**

Our findings suggest that endothelial dysfunction may be already present in children with 5 years or more elapsed since diagnosis, even in the absence of atherosclerotic structural changes. The decreased vasodilation response correlated with hyperglycemia.

## INTRODUCTION

Type 1 diabetes mellitus (T1DM) is currently one of the most common chronic diseases in childhood, displaying a high frequency of complications involving microvascular and macrovascular abnormalities (1).

Metabolic abnormalities characterizing T1DM, such as hyperglycemia and increased circulating free fatty acids, trigger molecular mechanisms that contribute to the development of endothelial dysfunction. These mechanisms include a decreased bioavailability of nitric oxide, increased oxidative stress, disturbances in intracellular signal transduction, and activation of advanced glycation end products (AGEs) (2).

Endothelial dysfunction, thought to be an early event in the structural changes of the atherosclerotic process, is characterized by abnormalities in the regulation of the lumen of the vessels, resulting in a blunted vasodilatory response (3). This vascular endothelial impairment results in increased production of inflammatory cytokines and augmented expression of cellular adhesion molecules and several other biologically active substances that contribute to the induction of a proinflammatory and prothrombotic state (4). These mechanisms represent an important step in the development of the initial atherosclerotic changes and subsequent complications in patients with T1DM (5).

Although the macrovascular complications of T1DM do not usually disclose major clinical manifestations during childhood and adolescence, evaluation with a reliable and noninvasive high-resolution ultrasound equipment allows an early diagnosis of endothelial dysfunction through the study of flow-mediated dilation (FMD) of the brachial artery. Additionally, the measurement of the carotid intima-media thickness (IMT) allows the detection of initial atherosclerotic alterations (6). FMD is defined as a reactive vasodilatation of the brachial artery after hyperemic stimulation (7) and when decreased, has a predictive value for future cardiovascular disease (8). Similarly, increased carotid artery width measured with the IMT technique is a risk factor for future atherosclerosis or suggestive of an already established atherosclerosis and an atherosclerotic process affecting peripheral, coronary, and femoral arteries. IMT is considered a strong predictor of vascular disease in high-risk individuals, such as those with T1DM (9).

The evaluation of children with T1DM using the FMD technique and IMT measurement at an early preclinical stage of the disease could allow the implementation of strategies to prevent or at least reduce the cardiovascular morbidity and mortality in diabetic children. Consequently, the aim of our study was to evaluate children with T1DM without evidence of microvascular or macrovascular complications for the presence of endothelial dysfunction and early vascular structural abnormalities applying the techniques of FMD and IMT.

## SUBJECTS AND METHODS

### Subjects

The study group comprised 31 children aged 6 to 12 years (mean age 8.36 ± 1.81 years) divided into two subgroups according to the time elapsed since the diagnosis of T1DM: subgroup 1, diagnosed within the previous 5 years (mean time from diagnosis 2.73 ± 1.11 years) and subgroup 2, diagnosed more than 5 years before the study (mean time from diagnosis 6.11 ± 1.28 years).

A total of 58 healthy children aged 6-12 years (mean 6.9 ± 1.77 years) comprised the control group. The diabetic children were recruited among patients attending the Pediatric Endocrinology Outpatient Clinic at the Brasilia University Hospital and the Pediatric Clinic of the Brasilia Children’s Hospital. Children in the control group were selected among dependents of the hospital’s employees. In order to minimize the influence of other factors on the endothelial function excluding those due to T1DM, we followed the criteria of inclusion and exclusion described in Table 1.

**Table 1:** Inclusion and exclusion criteria

Inclusion criteria for the T1DM children group
Absence of microvascular complications*

**Inclusion criteria for the control group**

Normal weight and height for age**
Fasting glucose < 100 mg/dL
HbA1c ≤ 5.6%
hsCRP < 0.3 mg/dL

**Exclusion criteria for both groups**

Onset of puberty***
Arterial hypertension****
Total cholesterol ≥ 200 mg/dL
HDL cholesterol < 45 mg/dL
LDL cholesterol ≥ 130 mg/dL
VLDL cholesterol ≥ 41mg/dL
Triglycerides ≥ 100 mg/dL
Hemoglobin < 11 g/dL
Hematocrit < 33%
Family history of primary dyslipidemia
Family history of premature death from CVD or stroke
Presence of acute infectious conditions
Chronic diseases, lasting 3 months or more, excluding T1DM
Continuous use of drugs, except insulin

T1DM: type 1 diabetes mellitus; HbA1c: glycated hemoglobin; hsCRP: high sensitive C-reactive protein; CVD: cardiovascular disease.

*All children underwent a detailed physical examination by a pediatric endocrinologist (that included assessment of blood pressure and skin sensitivity), specific serum and urinary biochemical tests (including a complete renal function and microalbuminuria), and complete ophthalmic evaluation. ** According to CDC growth charts (Kuczmarski and cols., 2002 [30]) adapted for Brazilian children (Silva and cols., 2010 [31]). *** Children not presenting Tanner stage M1P1 (females) or G1P1 (males) were excluded. **** Blood pressure was measured with suitable cuffs and classified according to systolic and diastolic curves, specific for each age, gender, and height, in agreement with the curves of the Second Task Force on Blood Pressure Control in Children (1996). High blood pressure was diagnosed when the percentiles of systolic and/or diastolic readings were above the 95^th^ percentile for age and sex, on at least three occasions. If the readings were between the 90^th^ and 95^th^ percentiles, the blood pressure was considered to be normal-high, and when below the 90^th^ percentile, it was considered normal.

The study was conducted according to the Declaration of Helsinki, and the Ethics Committee of the University of Brasilia School of Medicine approved the study protocol. Written informed consent was obtained from the legal guardians of the children.

### Study design and sample size calculation

The sample size was calculated with the software Epi Info, version 3.5.3. A minimum sample size of 66 children was obtained, considering a maximum error of 5% and a 95% confidence interval. The final sample comprised 31 patients with T1DM and 58 healthy controls, totaling 89 children.

### Ultrasound studies

The ultrasonographic studies were performed with ultrasound equipment model ACUSON X 300 (Siemens HG, Munich, Germany), equipped with a vascular software for two-dimensional (2D) and color images, color and spectral Doppler, internal monitor for echocardiography, and a high-frequency (8.9 MHz) vascular transducer (VF13-5). All images were obtained and evaluated by the same examiner and processed manually with an ultrasonic compass. Each child underwent both ultrasound techniques sequentially, always in the afternoon, in dimmed light, and at room temperature (23°C).

### Brachial artery FMD

Neither the consumption of high-fat foods, vitamin C, and stimulants beverages (*e.g.* caffeine), nor intense physical activity during the previous 24 hours was allowed before the FMD evaluation. All tests were preceded by blood pressure measurement after 10 minutes of rest, using a pneumatic sphygmomanometer with a cuff appropriate for the child’s upper limb size. Electrodes were applied for continuous electrocardiographic monitoring. In accordance with protocols suggested by Corretti and cols. and Barac and cols. (10,11), the children were placed comfortably in a supine position with the sphygmomanometer cuff placed on their left forearm over the brachial artery, 5 to 15 cm above the antecubital fossa. Initially, a baseline image of the left brachial artery in a longitudinal plane was obtained, and its quality was further optimized in a 2D mode. The image was subsequently expanded and recorded for 1 minute to allow later measurement of the larger artery diameter at rest (at the end of diastole, which coincides with the R wave of the QRS complex of the continuous ECG). The cuff was then inflated 50 mmHg above the systolic pressure (10,11) during 4 minutes (8). New images of the brachial artery were obtained 1, 3, 5, and 9 minutes after cuff deflation. After that, new images were obtained allowing measurement of the maximal diameter and the flow of the brachial artery. The arterial lumen diameter was defined as the distance between the intima of the far and near vessel walls. The dilatation was calculated by subtracting the lumen diameter at baseline from the maximal lumen diameter after ischemia and dividing the result by the lumen diameter at baseline (12). Results were expressed as a percentage FMD (%).

### Carotid artery IMT

All measurements were performed according to the protocol described by Järvisalo and cols. and Bots and cols. (13,14). Images of the right and left common carotid arteries were obtained in 2D mode with the subjects in a supine position with their heads lateralized, and the transducer positioned on the contralateral vessel at an approximate angle of 45°, about 1 to 2 cm proximal to the bifurcation of the carotid artery. The images of each artery were subsequently magnified to allow the measurement of the IMT of the posterior wall, at the end of the diastole (at the onset of the QRS complex). The IMT was defined as the distance between the lumen-intima and media-adventitia ultrasound interfaces of the carotid artery measured in mm (12).

### Statistical analysis

The results are shown as mean ± standard deviation. Statistical analyses were performed using the software GraphPad Prism, version 5.0 (GraphPad Software, San Diego, California, USA). Student’s *t* test or permutation tests (Kruskal-Wallis) were applied for comparisons with the control group. One-way ANOVA was used when applicable, and Dunnett’s post-test was used when diabetic children were compared with the children in the control group. To quantify the association between two variables, Pearson’s correlation coefficient was used. The data were considered statistically significant when *p* ≤ 0.05.

## RESULTS

### Characteristics of the groups

The demographic and laboratory profile of the diabetic and control groups are shown in Table 2. Both groups were similar regarding gender and age. The laboratory profiles showed statistical differences in levels of triglycerides and VLDL cholesterol between the groups, although the results were still within the normal reference values. As expected, the levels of glycated hemoglobin (HbA1c) and fasting glucose were significantly higher in the diabetic group.

**Table 2:** Laboratory and demographic data of the study population

Demographic parameters		Diabetics groups			*P* value

	Controls (n = 58)	T1DM (n = 31)	T1DM < 5 (n = 22)	T1DM ≥ 5	
Female	24 (41.4%)	12 (38.7%)	8 (36.4%)	4 (44.4%)	0.8068
Age	8.36 (±1.81)	9.06 (±1.77)	8.64 (±1.79)	10.11 (±1.27)	0.0817

**Laboratory parameters**		**Diabetic groups**			***P* value**

	**Controls**	**T1DM total**	**T1DM < 5**	**T1DM ≥ 5**	

Hemoglobin (mg/dL)	13.65 (±0.84)	13.70 (±0.90)	13.54 (±0.81)	14.10 (±1.03)	0.8060*
Hematocrit (%)	40.26 (±2.97)	40.30 (±2.32)	39.87 (±2.25)	41.36 (±2.26)	0.9402*
Glycated hemoglobin (%)	5.30 (±0.27)	9.04 (±1.64)	8.83 (±1.56)	9.57 (±1.79)	< 0.0001* 0.015** 0.018***
Total cholesterol (mg/dL)	166.95 (±22.14)	165.42 (±16.92)	165.27 (±17.53)	165.78 (±16.33)	0.7172*
Triglycerides (mg/dL)	63.80 (±16.7)	60.23 (±20.26)	57.95 (±18.35)	65.78 (±24.62)	0.0198*
HDL cholesterol (mg/dL)	51.47 (±9.70)	53.87 (±8.66)	51.59 (±6.73)	59.44 (±10.64)	0.2357*
LDL cholesterol (mg/dL)	101.95 (±18.06)	99.84 (±17.09)	102.59 (±16.71)	93.11 (±17.05)	0.5884*
VLDL cholesterol (mg/dL)	12.60 (±3.33)	14.40 (±4.84)	11.59 (±3.69)	13.22 (±4.79)	0.0179*
Fasting glucose (mg/dL)	84.40 (±9.03)	187.16 (±99.01)	194.14 (±103.94)	170.11 (±89.11)	< 0.0001* 0.0002** 0.002***

*P values were obtained by comparing the mean (± standard deviation [SD]) values obtained in each group, applying Student’s t test. ** P values were obtained by comparing the mean (±SD) values obtained in the control group versus those in the T1DM < 5 subgroup. *** P values were obtained by comparing the mean (±SD) values obtained in the control group versus those in the T1DM ≥ 5 subgroup applying Student’s t test, chi-square test, and Kruskal-Wallis test. All results are expressed as mean ± SD values.

Anthropometric data: percentiles (ranging from 50-85%) were normal for age and weight both in the diabetic and control groups. The mean body mass index (BMI) for the group of diabetic children was 16.9 kg/m^2^ and for nondiabetic children was 17.2 kg/m^2^. The mean weights in both groups were 31.5 kg and 33.4 kg, respectively, and the mean heights were 133.6 cm and 135.4 cm, respectively.

In order to establish if the disease duration influenced the results above, the diabetic group was further divided into two subgroups. The first subgroup comprised 22 children diagnosed with diabetes less than 5 years before the study (T1DM < 5, 14 males and 8 females, mean age 8.64 ± 1.79 years). The second group comprised 9 children diagnosed with diabetes more than 5 years before the study (T1DM ≥ 5, 5 males and 4 females, mean age 10.1 ± 1.27 years). When compared with the control group, both subgroups (T1DM < 5 and T1DM ≥ 5) continued to show significant *p* values for HbA1c (8.83 ± 1.56%, *p* = 0.015 and 9.57 ± 1.79%, *p* = 0.018, respectively) and fasting glucose (194.14 ± 103.94 mg/dL, *p* = 0.0002 and 170.11 ± 89.11 mg/dL, *p* = 0.002, respectively) when compared with the control group.

### Ultrasound studies

Results obtained with the brachial artery FMD technique are shown in Table 3.

**Table 3:** Ultrasonographic parameters showing the maximal percentage flow-mediated dilation (FMD) in the diabetic and control groups

Parameters analyzed		Diabetic groups			*P* value

	Controls	T1DM total	T1DM < 5	T1DM ≥ 5	
Mean brachial artery baseline diameter (mm)	2.59 (±0.13)	2.59 (±0.13)	2.58 (±0.13)	2.60 (±0.13)	0.887*
Max FMD (%)	11.45 (±2.86)	9.29 (±2.79)	10.21 (±2.70)	7.04 (±1.38)	0.0008* 0.078** 0.0001***
Max FMD time (min)	1 (±1.13)	1(±1.69)	1(±1.81)	1 (±1.41)	0.753*

T1DM: type 1 diabetes mellitus; FMD: flow-mediated dilation; max FMD (%): maximal FMD (%); max FMD time: time to the occurrence of the maximal FMD.

* P values were obtained by comparing the mean (± standard deviation [±SD]) values in the control group with those in the entire T1DM group, with the application of the Kruskal-Wallis test. ** P values were obtained by comparing the mean (±SD) values in the control group with those in the T1DM < 5 subgroup. *** P values were obtained by comparing the mean (±SD) values in the control group with those in the T1DM ≥ 5 subgroup, with the application of the Kruskal-Wallis test. All results are expressed in mean (±SD) values.

No significant differences were found in the percentage increase in the arterial FMD (%) between the control group (11.45 ± 2.86%) and subgroup T1DM < 5 (10.21 ± 2.70%, *p* = 0.078), but a significant difference was observed when the control group was compared with the subgroup T1DM ≥ 5 (7.04 ± 1.38%, *p* = 0.0001). The timing of the maximal dilation after stimulation did not change with the division into subgroups. There was no significant difference in the percentage of increase in FMD between genders (data not shown), both in the control group and diabetics subgroups in the four repeated measurements performed after post-occlusive reactive hyperemia. Similarly, no significant differences between groups were observed in the analysis of parameters related to the brachial artery, including systolic peak (SP), pulsatility index (PI), resistivity index (RI), maximum acceleration time (AT max), end diastolic speed (ED), and the ratio peak systolic velocity/peak diastolic velocity (S/D). The results from the measurements of the carotid arteries obtained with the IMT technique are shown in Table 4.

**Table 4:** Carotid artery intima-media thickness (IMT) measurements by the ultrasound technique in the diabetic and control groups

Parameters		Diabetic groups			*P* value

	Controls	T1DM total	T1DM < 5	T1DM ≥ 5	
IMT LCA mean	0.55 (± 0.04)	0.56 (± 0.06)	0.55 (± 0.05)	0.57 (± 0.08)	0.762* 0.737** 0.208***
IMT RCA mean	0.55 (± 0.05)	0.56 (± 0.04)	0.56 (± 0.04)	0.54 (± 0.05)	0.359* 0.185** 0.805***
Max IMT LCA and RCA (mm)	0.61 (± 0.06)	0.61 (± 0.05)	0.60 (± 0.04)	0.63 (± 0.07)	0.826* 0.628** 0.252***

IMT: intima-media thickness; LCA: left carotid artery; RCA: right carotid artery; Max: maximal.

* *P* values correspond to the comparison of the mean (± standard deviation [SD]) values in the entire T1DM group versus those in the control group. ** *P* values correspond to the comparison of the mean (±SD) values in the T1DM < 5 subgroup and those in the control group. *** *P* values correspond to the comparison of the mean (±SD) values in the T1DM ≥ 5 subgroup with those in the control group. The permutation test of Kruskal-Wallis was applied in the calculations. All results are expressed in mean (±SD) values.

### Correlation between the variables FMD (%) and HbA1c

**Figure 1:**
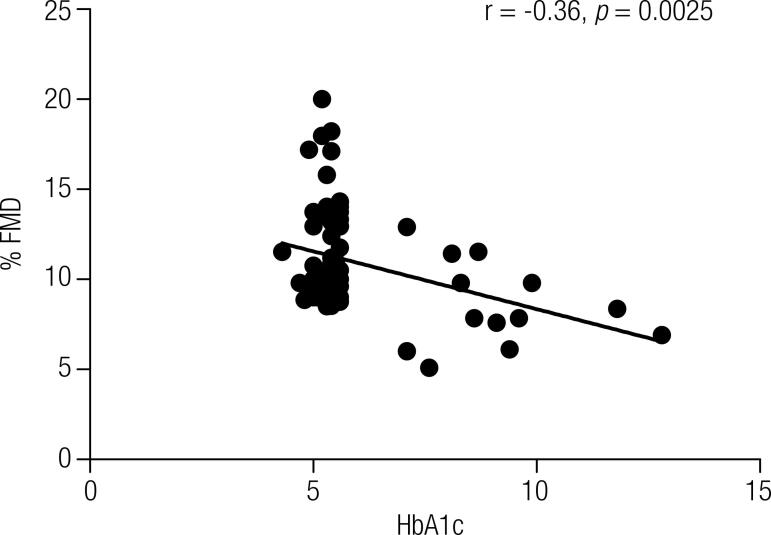
Pearson’s linear correlation scatter plot of the percentage of maximal flow-mediated dilatation (FMD %) versus glycated hemoglobin (HbA1c) levels.

**Figure 2:**
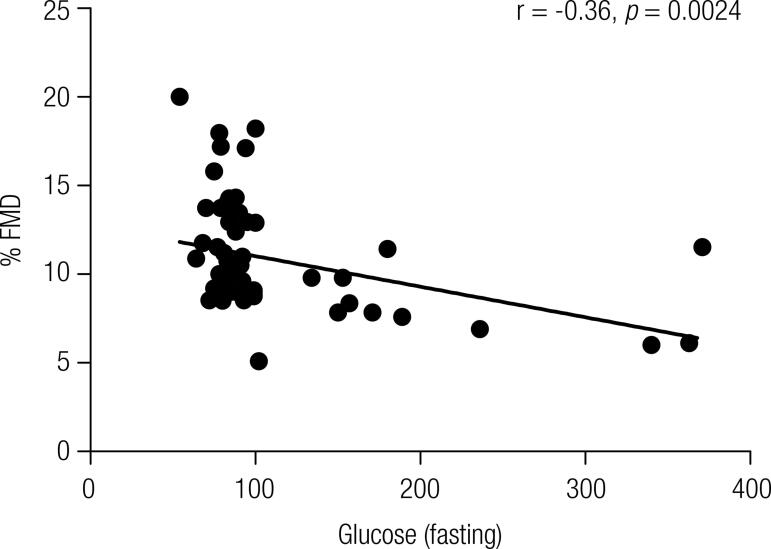
Pearson’s linear correlation scatter plot of the percentage of maximal flow-mediated dilation (FMD %) versus fasting glucose levels.

Pearson’s linear correlation coefficient, which evaluates the correlation between two measurable variables, was applied to determine the presence of a relationship between the FMD (%) and serum levels of HbA1c (Figure 1), and FMD (%) and fasting glucose (Figure 2). The test revealed a moderately negative correlation between FMD (%) and HbA1c (r = -0.36, *p* = 0.0025) and between FMD (%) and fasting glucose (r = -0.36, *p* = 0.0024) (Figure 2). As these correlations were negative, the coefficient suggested that the higher the level of HbA1c and fasting glucose, the lower the FMD (%), confirming our hypothesis.

## DISCUSSION

Macrovascular and microvascular complications are the main causes of morbidity and mortality in patients with diabetes mellitus, and their main trigger may be represented by dysregulation of the modulatory function of the vascular endothelium (15). It is an accepted fact that T1DM predisposes to premature atherosclerotic artery disease and there is evidence that the endothelial function impairment precedes the establishment of this pathological process (16).

The endothelial cells actively adjust the vascular tonus and reactivity in physiological and pathological conditions in response to mechanical stimuli and neurohumoral mediators with the release of a variety of factors leading to vascular constriction or relaxation. Additionally, these cells synthesize substances with a regulatory function on inflammation and hemostasis (3). Since the action of these cells can affect one or more functions simultaneously or sequentially, a gold-standard test to evaluate the presence of endothelial dysfunction has not yet been established (3). However, the endothelial function has been generally evaluated by changes in blood flow or measurement of blood vessel diameter in response to mechanical or chemical stimulation, measured invasively (by coronary catheterization) or noninvasively (by ultrasound technique) (3). The latter was the method used in our study.

Our results revealed significant differences in relation to fasting glucose and HbA1c levels between the diabetic patients’ groups (T1DM < 5 and T1DM ≥ 5) and the control group (Table 2), reflecting a suboptimal glycemic control in diabetic patients independent of the time from diagnosis. A lasting hyperglycemic state can affect the endothelial function due to alteration of molecular regulatory mechanisms of the nitric oxide synthesis and/or degradation (4) and the onset of oxidative stress inducing a deleterious effect on the endothelial cells (17). This process involves four metabolic pathways, with activation of protein kinase C (PKC), the formation of AGEs, overactivity of the hexosamine pathway, and increased flux of glucose through the polyol (sorbitol) pathway (1). Thus, the imbalance between the production of endothelium-derived factors impairs the vascular tonus and other physiological properties of the vascular endothelium (3), favoring vasoconstriction (18) and an inflammatory state (4), perpetuated by the long-lasting hyperglycemia (19). The results of Pearson’s correlation coefficients corroborate these claims in our population, suggesting a moderate association between decreased vasodilation and increased serum levels of HbA1c and fasting glucose (Figures 1 and 2, respectively).

On reviewing the FMD technique protocol, we found a disagreement among several authors regarding the degree of occlusion to be applied. Corretti and cols. (10) and Barac and cols. (11) advocate inflating the blood pressure cuff to ≥ 50 mmHg above the patient’s systolic pressure, while Shivalkar and cols. (8) recommend inflating the cuff to 100 mmHg above the systolic pressure. In our pediatric population, we chose to inflate the cuff to 50 mmHg above the systolic pressure, considering that there was a consensus in this regard between two of the authors and because this pressure was more comfortable for the children.

Another controversial point was the duration of the occlusion. Corretti and cols. (10) favored an occlusion duration of 5 minutes since changes in the vessel diameter are similar after 5 or 10 minutes and an occlusion for 5 minutes is more easily tolerated. However, in our experience, this recommendation was difficult to be carried out in our children, who showed considerable intolerance toward remaining with the cuff inflated 50 mmHg above the systolic pressure throughout the 5 minutes. Based in a previous study (8) that applied an occlusion duration of 4 minutes to adult patients obtaining good results, and considering that our patients were aged 6 to 12 years, we adopted this 4-minute occlusion time. This duration was well tolerated by our children and did not affect the test since the increase in the brachial artery diameter after cuff release occurs within 30 seconds to 5 minutes, and these changes in diameter are similar after 5 and 10 minutes (10).

The maximal dilatation response in our diabetic and control children was obtained 1 minute after the hyperemic stimulus (Table 3). Previous studies have suggested that the maximal increase in arterial diameter occurs approximately 60 seconds after cuff release (20-22). Our results are in accordance with those obtained by other authors, and the baseline brachial artery diameter was similar in all our groups (Table 3), thus eliminating any bias related to the baseline artery size since arteries with larger diameters show lower percentages of maximal dilation and vice versa (7).

In spite of FMD abnormalities in the T1DM ≥ 5 subgroup when compared with controls (Table 3), we could not find in our study IMT abnormalities in any of the diabetic subgroups (Table 4), which suggests the inexistence of atherosclerotic structural alterations that usually evolve after the advent of endothelial dysfunction.

Assessing our results and comparing our FMD and IMT findings with data reported in a previous study, we detected similar results in a group of diabetic children with a mean age of 14 years and a disease duration of 6 years. These children also failed to show vascular complications, although they presented impaired endothelial function assessed by FMD when compared with a control group (23). Similarly, a decreased FMD response was detected in a Finnish population with T1DM without microvascular complications, aged 7 to 14 years and with a mean disease duration of 4.4 ± 2.9 years. However, differently from our results, the subgroup with endothelial dysfunction in that study had significantly higher values of carotid IMT than the subgroup without endothelial dysfunction and the control group; no statistically significant differences were observed in the brachial artery baseline diameter, disease duration or HbA1c levels among diabetic children with and without endothelial dysfunction (6). In another study, Singh and cols. (24) also found no differences in carotid artery IMT between diabetic patients and control individuals, although the FMD detected was significantly lower in the diabetic group. These authors concluded that although the endothelial dysfunction appears during the first decade of T1DM onset, increased carotid IMT only occurs after a more extended time.

In another, more recent study, Eltayeb and cols. (25) assessed the endothelial function and myocardial alterations in pediatric patients with T1DM aged 5 to 16 years, without microvascular complications, and with a duration of 1-4 years from the initial diagnosis. These authors also reported a maximal FMD significantly lower and carotid IMT significantly higher in the diabetic group when compared with the control group. Similarly, a Turkish T1DM study conducted by Çiftel and cols. (27) in a group of children aged 7 to 16 years with a disease duration of at least 5 years from the diagnosis and no microvascular diabetic complications or additional cardiovascular risk showed a lower FMD and higher carotid IMT when comparing diabetic subjects with controls.

A recent Italian longitudinal study by Bruzzi and cols. (28) demonstrated in a group of children and adolescents aged 5 to 18 years and with a mean time elapsed from diagnosis of 4 years, that after a mean follow-up of 3 years, the endothelial function deteriorates sharply and that the male gender is considered a negative predictor of impaired FMD over time. Additionally, these authors reported that the maintenance of blood glucose levels near the normal range apparently does not avert the evolution of diabetic vasculopathy, suggesting the influence of intrinsic (genetic) and extrinsic (nutritional factors, physical inactivity) factors in the establishment of the endothelial damage.

The endothelial vasomotor activity is generally higher in females than males. Various hypotheses have been proposed to explain this fact: smaller body surface area, lower body mass index, and lower baseline diameter of the brachial artery in females when compared with males. Additionally, in prepubertal and adult subjects, estrogen can improve the endothelial function (29). No gender difference was detected in the vasodilatation response both in the T1DM and in the control group, probably due to similar baseline brachial artery diameters and lack of hormonal influence, since all children in our study were in the prepubertal stage.

This study is relevant for assessing the presence of endothelial dysfunction in T1DM children from an early age to the prepubertal stage, with a wide range of disease duration, and by noninvasive methods, further evidencing an association of diabetes with early structural atherosclerotic alterations. However, the study has some limitations. It does not clarify if the assessment of the endothelial function by the FMD technique and the laboratory determination of adhesion molecules and other inflammatory factors are clinically useful tools to stratify the cardiovascular risk in T1DM population groups or individual children. It also does not determine if an improvement in endothelial function would decrease the risk of development of future vascular atherosclerotic complications.

In conclusion, our study suggests that endothelial dysfunction without structural changes suggestive of atherosclerosis may appear in diabetic children with a history of 5 or more years of disease duration. In these children, a decreased vasodilation response correlates with a higher degree of hyperglycemia.

As the decreased reactivity of the brachial artery seems to improve with the modification of risk factors and drug treatment to reduce the cardiovascular risk, the acknowledgement of the presence of endothelial dysfunction in diabetic children allows the adoption of preventive strategies to mitigate the consequences of T1DM in this population, contributing to the reduction of the morbidity and mortality of the disease in adulthood.
